# Using multiple household food inventories to measure food availability in the home over 30 days: a pilot study

**DOI:** 10.1186/1475-2891-9-19

**Published:** 2010-04-15

**Authors:** Cheree Sisk, Joseph R Sharkey, William A McIntosh, Jenna Anding

**Affiliations:** 1 Intercollegiate Faculty of Nutrition, Texas A&M University, College Station, TX USA; 2 Program for Research in Nutrition and Health Disparities, School of Rural Public Health, Texas A&M Health Science Center, College Station, TX USA; 3 Department of Sociology, Texas A&M University, College Station, TX USA; 4 Department of Nutrition and Food Science, Texas A&M University, College Station, TX USA

## Abstract

**Background:**

The consumption of foods, especially by children, may be determined by the types of foods that are available in the home. Because most studies use a single point of data collection to determine the types of foods in the home, which can miss the change in availability when resources are not available, the primary objective of this study was to determine the extent to which the weekly availability of household food items changed over one month by 1) developing the methodology for the direct observation of the presence and amount of food items in the home; 2) conducting five in-home household food inventories over a thirty-day period in a small convenience sample; and 3) determining the frequency that food items were present in the participating households.

**Methods:**

After the development and pre-testing of the 251-item home observation guide that used direct observation to determine the presence and amount of food items in the home (refrigerator, freezer, pantry, elsewhere), two trained researchers recruited a convenience sample of 9 households (44.4% minority); administered a baseline questionnaire (personal info, shopping habits, food resources, and food security); and conducted 5 in-home assessments (7-day interval) over a 30-day period. Each in-home assessment included food-related activities since the last assessment, and an observational survey of types and amounts of foods present.

**Results:**

Complete data were collected from all 9 women (32.8 y ± 6.0; 3 married; 4 ± 1.6 adults/children in household; 4 received food assistance; and 6 had very low food security) and their households. Weekly grocery purchases (place, amount, and purpose) varied from once (n = 1) to every week (n = 5); 4 used fast food 2-3 times/wk for 4 weeks. The weekly presence and amounts of fresh and processed fruits and vegetables and dairy varied.

**Conclusions:**

The feasibility of conducting multiple in-home assessments was confirmed with 100% retention of participants through 5 in-home assessments, which paid particular attention to the intra-monthly changes in household availability in type and amount of foods. This study contributes to research on home food availability by identifying the importance of multiple measures, presence of certain foods in the home, and the feasibility of comprehensive in-home assessments.

## Background

Obesity and overweight continue to present broad-scale problems throughout the world, with high obesity rates in the United States among African American and Hispanic populations [1-6], persons with low income and educational attainment [7], and individuals living in rural areas [8,9]. There is very little argument that food choice, which is influenced by food available in the home, affects nutritional health [10]. In fact, Rasmussen and colleagues report home food availability as one of the most important determinants of eating behavior [11]. Further, household food supplies are considered an intermediate step between community or neighborhood retail food sources and individual intake [12].

The home food environment supplies more than 70% of the food, by weight, eaten by Americans, and has been strongly associated with 75% of energy intake and overall food consumption [10,13-19]. Understanding and changing the home food environment to decrease the consumption of unhealthy foods requires an accurate measurement of the foods that are available in the home. Assessing the presence of various foods in the home, including both healthful and less healthful, may provide understanding of what foods are available for home consumption and insight needed in order to assess dietary behavior [20-23]. Studies have shown that foods found in pantries are indicators of actual food consumption, and there has been debate that availability influences food intake [24-26].

A variety of methods for assessing home food supplies have been developed and used in recent years. Two general approaches have been used to document the presence of food items in the home; namely, grocery store receipts and household food inventories [1,2,12,14,15,17,22-25,27-39]. They are similar in that they attempt to measure the presence of certain food items in the home; however, frequency of observations, the types of food being measured, and method of the data collection vary [12].

Inventories of household food supplies (HFI), which assess the presence of a wide range of food items in the home, may be an appropriate method for documenting the home food environment [34]. Open inventories and predefined inventories are two of the more common household food inventory methods [40]. Open inventories are customarily conducted by trained researchers who travel to a subject's home and record all foods present in the home [23,36,41]. In many cases, household inventories have focused on a particular food category such as fruit and vegetables, fats, soft drinks, or cancer preventing foods, using a predefined checklist to document whether a food was present in the home [1,14,15,21,24,34,35,38,39]. The method of data collection varies from direct observation in the home by trained researchers (considered the "gold standard") to telephone-administered or mailed self-report by participants [12]. Cullen and colleagues concluded that self-reported data are subject to possible attention, comprehension, memory, and recording errors [21]. Self-reported error is especially of concern in studies conducted outside of the home, where participants are asked to recall the types of food items present in their homes when they are in a location other than their homes [14,24,25,28,42].

The number of times an inventory should be conducted in order to describe usual availability has yet to be determined. Still, most studies choose a single point of data collection in conducting an inventory of household food supplies [1,12,15,17,21,23-25,28,35,41,42]. Unfortunately, a single point of data collection may miss the influence of intra-monthly variability on food supplies due to income cycles, purchasing behavior, limitations in storage and refrigeration, family events, and other factors. Therefore, one measurement may not accurately represent the foods usually available in the home. Similar to a single dietary recall, which would not capture variations in dietary habits, a single food inventory does not capture variation in home food availability [43]. To date, there are a limited number of household food inventory studies that visit the home on more than one occasion [30,44-47]. Baranowski and colleagues measured the availability of fruits, juice, and vegetables on three different occasions over the course of one year [47]. Kendall and colleagues collected household food inventory data two times with a three-week interval between visits [46]. Similarly, Weinstein and colleagues collected food inventory data with the UPC scanner three times over four weeks (no more than one time per week) [30]. It is not known how many times or the frequency multiple observations should be conducted in order to obtain a more accurate representation of what is usually in the home.

Since little is known how household food supplies vary over a month [12], this pilot study expands our understanding household food availability by: 1) developing the methodology for the direct observation of the presence and amount of food items in the home; 2) conducting five in-home household food inventories over a thirty-day period in a small convenience sample; and 3) determining the frequency that food items were present in the participating households.

## Methods

### Participants

Eligibility for inclusion in the HFI was limited to women with at least one child under the age of eighteen living in the same household. Eleven women were recruited from a local child care center, supermarket, and community action agency through flyers and word-of-mouth. Two were excluded from the study after multiple failed attempts to schedule the first home visit, leaving a sample of nine women. The study was completed in July-August, 2008. Participants received a cash incentive for participation in the study, which was distributed at the end of the study; $15 for each of the in-home assessments (HFI and surveys). Informed consent was obtained from all participants, and the study was approved by the Institutional Review Board at Texas A&M University.

### Baseline Questionnaire

During the first home visit, an interviewer-administered questionnaire was completed; and included the following sections: 1) sociodemographic characteristics, 2) food-related activities, and 3) food security. **Sociodemographic characteristics **included participant's age, years of completed education, race/ethnicity, marital status, number of adults and children residing in the household, ages of children, household income in 2007, frequency of income payments, automobile ownership, and nutrition program participation (e.g., Supplemental Nutrition Assistance Program [SNAP], Women, Infants, and Children Nutrition Program [WIC], School Breakfast Program, and School Lunch Program). **Food-related activities **included one-way distance to the store where most of the household's groceries are purchased; frequency of shopping at this store (weekly, bi-weekly, monthly, or less than once a month); amount spent on groceries; days since the last food shopping and amount spent; and frequency of prepared meals purchased from a fast or full-service restaurant and consumed at home or at the restaurant. **Food security **was measured using the U.S. Household Food Security Survey Module: Six-Item Short Form [48]. During the 12 months prior to the first home visit, food security status was operationalized from the following food security risk situations: purchased food did not last and money was not available to get more; could not afford to eat balanced meals; adults in the household cut the size of meals or skipped meals because there wasn't enough money for food; adults eat less than should eat because there wasn't enough money for food; and were hungry and did not eat because couldn't afford enough food. The first three questions also asked the frequency the situation occurred (often, sometimes, or never). If the participant answered often or sometimes, they were then asked whether or not this happened every month, 1-2 months, or some months. Scores were calculated to classify households as food secure (score = 0), marginal food security (score = 1), low food security (score = 2-4), and very low food security (score = 5-6).

### Household Food Inventory (HFI)

Household food supplies were inventoried using a HFI instrument that included 251 items and was modified from a 171-item self-reported shelf inventory survey used in low-income families [29]. The HFI was reviewed by community dietitians in the area; food items were added to include canned and frozen fruit and vegetables and regional food items. Prepared foods from full-service or fast food restaurants were not included. In addition, the instrument was modified to facilitate direct observation of the presence and amount of specific food items. The HFI consisted of the following categories: fresh, canned, and frozen vegetables; fresh, canned, and frozen fruit; canned fruit; canned vegetables; legumes; dairy (milk, yogurt, and cheese); fresh, canned, and frozen meat, poultry, seafood (fresh or frozen) and other protein foods; cereals, breads, and tortillas; chips, crackers, and other snacks; frozen desserts (e.g., ice cream and popsicles); frozen foods (e.g., pizza, tacos or burritos, entrees, breakfast items, and French fries); beverages; and oils and other fats. Amounts were determined by a count of the number of items in the case of whole fresh fruit and vegetables, labeling of bottled, canned, or prepackaged foods, and estimation of previously opened or sliced food items.

### Follow-up Questionnaire

A follow-up questionnaire was administered during home visits 2, 3, 4, and 5 to identify food-related activities that occurred since the prior home visit. The following questions were included: 1) did you purchase groceries (where, how much was spent, type of purchase, and method of transportation); 2) did you eat at a fast food restaurant (and frequency); 3) did you eat at a restaurant (and frequency); and 4) did you purchase food prepared elsewhere to eat at home (and frequency). Frequency responses included once, 2-3 times, 4-5 times, > 5 times, or does not apply.

### Data Collection

Data were collected in each participant's home by a trained researcher team during five home visits, which were scheduled to occur over thirty days; each home visit was scheduled to occur approximately 6-7 days after the prior home visit. The study was conducted during July, August, and early September 2008. During the first visit, the baseline questionnaire was administered, an assessment of kitchen appliances was completed, and a comprehensive inventory of household food supplies was conducted in refrigerators, freezers, cabinets, and storage areas and on counter-tops. Participants were asked to identify all areas that contained any food or beverage items. Photographs were taken of the appliances and all of the places where food was stored in the home. During home visits 2-5, a follow-up questionnaire was administered; a complete household food inventory was assessed; and photographs were taken of food supplies. The researchers developed a "call out" method where one would call out the presence and amount of each food item while the other researcher recorded the information. The research team would randomly re-inventory an area to verify the initial count.

### Data Analysis

Survey and household food inventory data were entered into a relational database (Microsoft Office Access 2007); descriptive statistics were calculated for mean and frequency of sample characteristics, food-related activities, food security, and the presence of individual food items, using Stata statistical software release 9 (Stata Corp., College Station, TX).

## Results

All nine participants completed all aspects of the study; there were no drop-outs after the first home visit. All of the appointments were conducted with rescheduling required on two occasions. One participant's child was ill, which required a two-week interval between her second and third appointments. All other appointments were conducted with 5-7 days interval between visits; and all 9 women completed all five in-home assessments. On average the first household food inventories required 45 minutes to complete; the average time required for the remaining four HFI was 30 minutes.

Characteristics of the participating women and their households are shown in Table 1. Four were non-Hispanic white; one was African American and four were Hispanic. About two-thirds reported a household income ≤$25,000. Household composition (combined adults and children) ranged from three (*n *= 4) to eight; 4 households included 5-8 adults and children. Three households did not participate in any supplemental nutrition program; six households participated in at least two nutrition programs (data not shown).

**Table 1:** Characteristics of All Participants from Baseline Questionnaire (*n *= 9)

	Mean ± SD (range)	% (n)
Age	32.8 ± 6.0 (23-41)	
Education, y	13.4 ± 3.9 (8-20)	
Race/ethnicity		
Minority		55.6 (5)
Marital status		
Married		33.3 (3)
Household composition		
Adults	2.1 ± 0.9 (1-4)	
Children	2.2 ± 1.6 (1-6)	
Total adults and children	4.3 ± 1.6 (3-8)	
Household income (in thousands)/y		
<$10		11.1 (1)
$10-$15		11.1 (1)
$16-$19.9		22.2 (2)
$20-$25		22.2 (2)
$30-$35		11.1 (1)
>$50		22.2 (2)
Frequency of income		
Weekly		22.2 (2)
Bi-weekly		44.4 (4)
Monthly		33.3 (3)
Car ownership		77.8 (7)
Nutrition program participation		
Supplemental Nutrition Assistance Program (SNAP)		44.4 (4)
Women, Infants, and Children (WIC) Program		22.2 (2)
Free school breakfast		22.2 (2)
Free or reduced school lunch		55.6 (5)

Although one-third (*n *= 3) were considered food secure, 44.4% (*n *= 4) were classified as having very low food security. Three households purchased groceries within seven days of all five assessments and four households for four assessments. Among these eight participants, six considered no more than one shopping occasion to be major. Although six participants consumed at least one fast food meal within seven days of 4-5 of the assessments, only two participants reported purchasing fast food or other prepared foods for home consumption prior to 4 assessments. Total expenses for groceries (sum of grocery experiences within seven days of all home assessments) was <$300 for three households, $300-$420 for five households, and >$450 for one household.

The number of household inventories in which individual fresh fruit and vegetables and overall variety were present varied (Table 2). Only three households had fresh fruit or vegetables at all five assessments; 44% of the households had no fruit or vegetables on 1-4 occasions. Weekly presence of fresh fruit was least consistent among very low food secure households; the presence of fresh vegetables was inconsistent among very low food secure and food secure households (data not shown). Apples and bananas were the most frequently observed fresh fruit; however, the amount of apples present in households with apples ranged from 1-14 apples (data not shown). In households with bananas, the number ranged from 1-10 bananas. Household availability of canned fruit and vegetables and legumes (canned and dry) can be found in Table 3. Seven households had no more than one type of canned fruit (in heavy syrup, light syrup, or juice) at any assessment; and the amount present varied in three households (data not shown). Although the most common canned vegetables were tomatoes, green beans, green peas, carrots, and corn, their presence was not consistent throughout the month. Further, the amount of canned tomatoes and green beans varied within six households; and in households with canned corn, the amount remained constant throughout the month.

**Table 2:** Percentage of Participants with Fresh Fruit and Vegetables Present During Five Household Food Inventories (*n *= 9)

	Number of Household Inventories in Which Foods Were Present					
	*5*	*4*	*3*	*2*	*1*	*0*
***Fresh fruit***						
Apples	11.1 (1)	11.1 (1)	11.1 (1)	11.1 (1)	22.2 (2)	33.3 (0)
Bananas	11.1 (1)	0	33.3 (3)	22.2 (2)	22.2 (2)	11.1 (1)
Grapes	22.2 (2)	11.1 (1)	0	11.1 (1)	11.1 (1)	44.4 (4)
Oranges	22.2 (2)	11.1 (1)	0	0	11.1 (1)	55.6 (5)
Peaches	0	11.1 (1)	22.2 (2)	22.2 (2)	0	44.4 (4)
Pears	11.1 (1)	0	11.1 (1)	11.1 (1)	0	66.7 (6)
Plums	0	0	0	33.3 (3)	11.1 (1)	55.6 (5)
Strawberries	22.2 (2)	0	0	11.1 (1)	11.1 (1)	55.6 (5)
Watermelon	11.1 (1)	0	22.2 (2)	0	22.2 (2)	44.4 (4)
*Variety*^a^						
0	0	22.2 (2)	0	11.1 (1)	11.1 (1)	55.6 (5)
1-2	0	22.2 (2)	0	11.1 (1)	33.3 (3)	33.3 (3)
≥3	33.3 (3)	0	11.1 (1)	11.1 (1)	11.1 (1)	33.3 (3)
***Fresh vegetables***						
Broccoli	0	0	11.1 (1)	0	11.1 (1)	77.8 (7)
Carrots	11.1 (1)	0	22.2 (2)	11.1 (1)	11.1 (1)	44.4 (4)
Celery	11.1 (1)	0	0	0	0	88.9 (8)
Corn	0	11.1 (1)	0	0	11.1 (1)	77.8 (7)
Cucumber	0	22.2 (2)	0	11.1 (1)	0	66.7 (6)
Greens	0	0	11.1 (1)	11.1 (1)	0	77.87 (7)
Lettuce	11.1 (1)	11.1 (1)	33.3 (3)	11.1 (1)	22.2 (2)	11.1 (1)
Potato	0	33.3 (3)	0	33.3 (3)	0	33.3 (3)
Squash	0	0	0	0	22.2 (2)	77.8 (7)
Tomato	11.1 (1)	44.4 (4)	0	22.2 (2)	11.1 (1)	11.1 (1)
*Variety*^b^						
0	0	11.1 (1)	0	0	33.3 (3)	55.6 (5)
1-2	0	22.2 (2)	0	11.1 (1)	33.3 (3)	33.3 (3)
≥3	33.3 (3)	11.1 (1)	11.1 (1)	11.1 (1)	11.1 (1)	22.2 (2)

^a ^Number of different types of fresh fruit (0, 1-2, and 3 or more)

^b ^Number of different types of fresh vegetables (0, 1-2, and 3 or more)

**Table 3:** Percentage of Participants with Can Fruit and Vegetables, Frozen Vegetables and Legumes Present During Five Household Food Inventories

	Number of Household Inventories in Which Foods Were Present					
	*5*	*4*	*3*	*2*	*1*	*0*
***Can fruit***						
Apples	22.2 (2)	11.1 (1)	0	11.1 (1)	0	55.6 (5)
Mixed fruit	11.1 (1)	22.2 (2)	0	0	0	66.7 (6)
Oranges	11.1 (1)	0	0	0	0	0
Peaches	0	22.2 (2)	0	0	0	77.8 (7)
Pears	11.1 (1)	0	0	0	0	88.9 (8)
Pineapple	22.2 (2)	11.1 (1)	0	0	11.1 (1)	55.6 (5)
***Can vegetables***						
Carrots	33.3 (3)	11.1 (1)	11.1 (1)	0	11.1 (1)	33.3 (3)
Corn	0	33.3 (3)	11.1 (1)	11.1 (1)	11.1 (1)	33.3 (3)
Greens	11.1 (1)	11.1 (1)	11.1 (1)	22.2 (2)	0	44.4 (4)
Green beans	33.3 (3)	11.1 (1)	11.1 (1)	11.1 (1)	11.1 (1)	22.2 (2)
Green peas	22.2 (2)	0	11.1 (1)	11.1 (1)	33.3 (3)	22.2 (2)
Mixed	44.4 (4)	0	11.1 (1)	0	0	44.4 (4)
Potato	0	11.1 (1)	0	0	11.1 (1)	77.8 (7)
Tomato	66.7 (6)	11.1 (1)	22.2 (2)	0	0	0
Yams	0	0	0	0	11.1 (1)	88.9 (8)
***Frozen vegetables***						
Broccoli	22.2 (2)	11.1 (1)	11.1 (1)	0	0	55.6 (5)
Corn	11.1 (1)	0	0	0	11.1 (1)	77.8 (7)
Greens	11.1 (1)	0	0	0	0	88.9 (8)
Peas	22.2 (2)	0	0	0	0	77.8 (7)
Green beans	0	11.1 (1)	11.1 (1)	0	0	77.8 (7)
Mixed	11.1 (1)	11.1 (1)	0	11.1 (1)	22.2 (2)	44.4 (4)
Potatoes	0	0	11.1 (1)	0	0	88.9 (8)
FF potatoes^a^	11.1 (1)	0	11.1 (1)	0	22.2 (2)	55.6 (5)
Tomatoes	0	0	0	0	22.2 (2)	77.8 (7)
***Legumes***						
Beans (dry)	33.3 (3)	11.1 (1)	22.2 (2)	11.1 (1)	11.1 (1)	11.1 (1)
Can beans	44.4 (4)	22.2 (2)	0	0	11.1 (1)	22.2 (2)
Lentils	0	0	11.1 (1)	11.1 (1)	11.1 (1)	66.7 (6)
Beans (sauce)	22.2 (2)	22.2 (2)	22.2 (2)	11.1 (1)	11.1 (1)	11.1 (1)
Refried beans	0	11.1 (1)	0	0	11.1 (1)	77.8 (7)

^a ^French fries

Household availability of dairy products can be found in Table 4. Two households had whole milk and one had low fat milk on all five occasions (and the amount varied from week-to-week); 44%-55% had whole or low fat milk present on 1-3 inventory occasions, with different amounts at each inventory (data not shown). In addition, yogurt and cheese were not consistently available. Table 5 indicates that meats, poultry, and seafood were not consistently available. The availability of cereals, breads, crackers, prepared desserts, noodles, and rice can be found in Table 6. White bread and sweetened cereals were found in most homes. In two-thirds of the homes white bread was present on 4-5 occasions; and in all households sweetened breakfast cereal was available on 4-5 occasions (with varying amounts from week-to-week). Unsweetened dry cereal was available in 77.8% of households; however, they were part of the household food supply on 1-3 occasions in 44.4% of households. Tortillas and biscuits were present on 1-3 occasions in 4-5 households; prepared desserts (e.g., donuts or regular cookies) were available during all 5 assessments in one household. With the exception of pasta, noodles and rice were not usually present. Table 7 shows the frequency of availability of chips, snacks, and frozen desserts; regular chips were available in all households on at least 4 occasions; the presence of other snack foods and regular ice cream varied. Table 8 shows the inconsistent availability of beverages.

**Table 4:** Percentage of Participants with Dairy Present During Five Household Food Inventories

	Number of Household Inventories in Which Foods Were Present					
	*5*	*4*	*3*	*2*	*1*	*0*
***Milk***						
Whole	22.2 (2)	11.1 (1)	11.1 (1)	11.1 (1)	22.2 (2)	22.2 (2)
Low fat	11.1 (1)	22.2 (2)	33.3 (3)	22.2 (2)	0	11.1 (1)
***Yogurt***						
Regular	0	11.1 (1)	0	11.1 (1)	33.3 (3)	44.4 (4)
Low fat	0	22.2 (2)	22.2 (2)	0	0	55.6 (5)
***Cheese***						
Cheese spread	11.1 (1)	11.1 (1)	0	11.1 (1)	33.3 (3)	33.3 (3)
Regular	55.6 (5)	22.2 (2)	22.2 (2)	0	0	0
Low fat	11.1 (1)	0	0	22.2 (2)	11.1 (1)	55.6 (5)

**Table 5:** Percentage of Participants with Meat/Poultry/Seafood and Other Protein Foods Present During Five Household Food Inventories

	Number of Household Inventories in Which Foods Were Present					
	*5*	*4*	*3*	*2*	*1*	*0*
Beef - regular	55.6 (5)	11.1 (1)	22.2 (2)	0	0	11.1 (1)
Pork						
Regular	11.1 (1)	0	33.3 (3)	33.3 (3)	0	22.2 (2)
Sausage	11.1 (1)	0	0	44.4 (4)	22.2 (2)	22.2 (2)
Bacon	44.4 (4)	11.1 (1)	11.1 (1)	11.1 (1)	11.1 (1)	11.1 (1)
Hot dogs						
Beef/pork	11.1 (1)	22.2 (2)	11.1 (1)	0	33.3 (3)	22.2 (2)
Turkey/chicken	0	0	22.2 (2)	11.1 (1)	11.1 (1)	55.6 (5)
Corn dogs	0	11.1 (1)	0	0	22.2 (2)	66.7 (6)
Lunch meat						
Ham/bologna	0	44.4 (4)	11.1 (1)	11.1 (1)	11.1 (1)	22.2 (2)
Salami	0	0	11.1 (1)	11.1 (1)	33.3 (3)	44.4 (4)
Miscellaneous	0	0	11.1 (1)	0	11.1 (1)	77.8 (7)
Chicken						
Breast	33.3 (3)	22.2 (2)	11.1 (1)	11.1 (1)	11.1 (1)	11.1 (1)
Whole/pieces	0	22.2 (2)	222.2 (2)	33.3 (3)	22.2 (2)	0
Breaded	11.1 (1)	11.1 (1)	11.1 (1)	22.2 (2)	22.2 (2)	22.2 (2)
Canned	11.1 (1)	22.2 (2)	22.2 (2)	11.1 (1)	11.1 (1)	22.2 (2)
Turkey	33.3 (3)	0	11.1 (1)	22.2 (2)	0	33.3 (3)
Fish						
Not breaded	11.1 (1)	11.1 (1)	11.1 (1)	11.1 (1)	11.1 (1)	44.4 (4)
Breaded	0	0	11.1 (1)	0	22.2 (2)	66.7 (6)
Canned fish	55.6 (5)	0	33.3 (3)	11.1 (1)	0	0
Eggs						
Whole	88.9 (8)	11.1 (1)	0	0	0	0
Substitute	11.1 (1)	0	0	0	0	88.9 (8)
Peanut butter						
Regular	66.7 (6)	11.1 (1)	11.1 (1)	11.1 (1)	0	0
Reduced fat	0	0	11.1 (1)	11.1 (1)	11.1 (1)	66.7 (6)

**Table 6:** Percentage of Participants with Cereals, Breads, Crackers, Prepared Desserts, Noodles, and Rice Present During Five Household Food Inventories

	Number of Household Inventories in Which Foods Were Present					
	*5*	*4*	*3*	*2*	*1*	*0*
Dry Cereal						
Unsweetened	33.3 (3)	0	11.1 (1)	11.1 (1)	22.2 (2)	22.2 (2)
Sweetened	66.7 (6)	33.3 (3)	0	0	0	0
Oatmeal	66.7 (6)	11.1 (1)	0	0	0	22.2 (2)
Bread						
White	55.6 (5)	11.1 (1)	22.2 (2)	0	0	11.1 (1)
Whole wheat	33.3 (3)	11.1 (1)	0	0	0	55.6 (5)
Tortillas						
Corn	22.2 (2)	0	0	22.2 (2)	33.3 (3)	22.2 (2)
Flour	22.2 (2)	0	22.2 (2)	11.1 (1)	11.1 (1)	33.3 (3)
Biscuits	22.2 (2)	0	33.3 (3)	11.1 (1)	0	33.3 (0)
Crackers						
Regular	44.4 (4)	33.3 (3)	22.2 (2)	0	0	0
Low fat	0	11.1 (1)	0	0	11.1 (1)	77.8 (7)
***Prepared Desserts***						
Donuts	11.1 (1)	11.1 (1)	11.1 (1)	22.2 (2)	0	44.4 (4)
*Pan dulce*	0	11.1 (1)	0	0	22.2 (2)	66.7 (6)
Cookies						
Regular	11.1 (1)	33.3 (3)	0	11.1 (1)	33.3 (3)	11.1 (1)
Reduced fat	0	0	0	0	0	100 (9)
***Noodles and Rice***						
Pasta	77.8 (7)	11.1 (1)	11.1 (1)	0	0	0
Ramen	11.1 (1)	22.2 (2)	11.1 (1)	22.2 (2)	0	33.3 (3)
Rice-A-Roni	11.1 (1)	22.2 (2)	0	0	33.3 (3)	33.3 (3)
Rice						
White	33.3 (3)	22.2 (2)	11.1 (1)	0	0	33.3 (3)
Brown	0	11.1 (1)	0	0	11.1 (1)	77.8 (7)
Hamburger helper	55.6 (5)	11.1 (1)	11.1 (1)	0	0	22.2 (2)

**Table 7:** Percentage of Participants with Chips, Snacks, and Frozen Desserts Present During Five Household Food Inventories

	Number of Household Inventories in Which Foods Were Present					
	*5*	*4*	*3*	*2*	*1*	*0*
Chips						
Regular	55.6 (5)	44.4 (4)	0	0	0	0
Baked	22.2 (2)	22.2 (2)	0	0	11.1 (1)	44.4 (4)
Pork skins	0	0	11.1 (1)	11.1 (1)	11.1 (1)	66.7 (6)
Pretzels	11.1 (1)	0	11.1 (1)	0	11.1 (1)	66.7 (6)
Pop corn	22.2 (2)	11.1 (1)	11.1 (1)	0	0	55.6 (5)
Nuts^a^	22.2 (2)	11.1 (1)	11.1 (1)	22.2 (2)	22.2 (2)	11.1 (1)
Candy	22.2 (2)	22.2 (2)	0	22.2 (2)	22.2 (2)	11.1 (1)
Granola bars	33.3 (3)	11.1 (1)	11.1 (1)	0	11.1 (1)	33.3 (3)
Pop tarts	22.2 (2)	0	22.2 (2)	0	11.1 (1)	44.4 (4)
***Frozen Desserts***						
Ice cream						
Regular	22.2 (2)	22.2 (2)	11.1 (1)	11.1 (1)	22.2 (2)	11.1 (1)
Low fat	11.1 (1)	11.1 (1)	0	0	0	77.8 (7)
Popsicles	66.7 (6)	0	22.2 (2)	11.1 (1)	0	0

^a ^Nuts of any kind

**Table 8:** Percentage of Participants with Beverages Present During Five Household Food Inventories

	Number of Household Inventories in Which Foods Were Present					
	*5*	*4*	*3*	*2*	*1*	*0*
Tea						
Sugar	0	0	44.4 (4)	11.1 (1)	11.1 (1)	33.3 (3)
Soda						
Regular (sugar)	22.2 (2)	11.1 (1)	0	44.4 (4)	11.1 (1)	11.1 (1)
Low sugar	22.2 (2)	0	0	0	11.1 (1)	66.7 (6)
100% fruit juice	33.3 (3)	0	11.1 (1)	0	22.2 (2)	33.3 (3)
Fruit drinks	11.1 (1)	11.1 (1)	11.1 (1)	11.1 (1)	33.3 (3)	22.2 (2)
Drink concentrate						
Regular sugar	11.1 (1)	11.1 (1)	11.1 (1)	22.2 (2)	22.2 (2)	22.2 (2)
Low sugar	22.2 (2)	22.2 (2)	0	11.1 (1)	0	44.4 (4)
Sports drinks	0	11.1 (1)	0	0	33.3 (3)	55.6 (5)

## Discussion

The availability of foods in the home is one of the factors that may influence the decisions individuals make with regard to food choice and consumption in the home. This is particularly important since the type of foods individuals consume affects their overall health and well being [49]. There are a number of factors that may influence household food availability, such as household composition, access to food outlets, household income, transportation, income/resource cycle, and refrigeration/storage facilities. This study examined the availability of food items in the home, paying particular attention to the changes in availability that occur throughout the month. This is apparently the first study to directly observe and document the weekly presence of the type and amount of foods over the course of one month. This study contributes to research on home food availability by identifying the importance of multiple measures, presence of certain foods in the home, and the feasibility of conducting multiple in-home assessments. The results of this pilot study may be applied to household dietary behaviors in the prevention and management of obesity, type 2 diabetes, cardiovascular diseases, and hypertension, and to characterize the overall nutrition climate in households.

Although researchers recognize the importance of documenting the availability of food items in the home, primarily through a single household food inventory (HFI) [1,12,15,17,21,24,25,28,35,41], little has been reported about the intra-monthly changes in household food supplies, which may be expected due to income cycles, grocery store trips, competing demands for resources, and family events. Much like dietary recalls, this variability is ignored when only one assessment is conducted, which may result in an inaccurate description of food items available for consumption. The primary objective of this pilot study was to determine the extent to which the availability of household food items changed over one month by 1) modifying an existing household food inventory instrument; 2) determining the feasibility of recruiting and retaining a convenience sample of households into a study that involved five in-home assessments over one month; and 3) determining the frequency that food items were present in the participating households.

Using direct observation methodology, which is considered more accurate than self-reported data [21], this study suggests that a single household assessment may be inadequate in describing the usual presence of food items in the home. It was evident that several food categories changed weekly. For example, individual and variety of fresh fruit and vegetables, milk, canned vegetables, and processed meats (e.g., hot dogs and lunch meat) were seldom observed during more than three of the five HFI, or the amounts present varied from week-to-week. Foods like canned fruits were seldom present; and in households with canned fruit, the amount did not vary from week-to-week. By simply going into the home on one occasion, we would not have captured "usual" availability. The weekly variation in all food products suggests the importance of conducting multiple in-home assessments in order to get an accurate representation of home food availability. Not only did the amount of food vary from week to week, but the types of foods present in the home varied as well

For the most part, previous household food inventory studies focused on a limited number of food categories, used predefined inventories, and did not record the amount of food present [1,14,15,21,34,35,38,39]. The present study used a comprehensive, predefined inventory that assessed a broad range of food groups to capture variation in all foods.

One-time HFIs received criticism in the past for only capturing a "cross-sectional snapshot" of what is usually in the home, and not taking into consideration away-from-home foods [12]. In response to that criticism, this study administered a short questionnaire at each in-home assessment to determine the number and type of places where food was purchased since the previous assessment. This provided insight into away-from-home food purchases and the weekly amount spent on grocery purchases. The frequency of grocery store trips varied with each individual. Participants who did not purchase groceries on a regular basis had less food at certain times of the month. Interestingly, the four households that did not purchase groceries on a weekly basis all purchased fast food at least once every two weeks. One particular household did not purchase groceries every week, but consumed fast food 2-3 times each week. In addition, the questionnaire addressed underlying issues that may have affected food purchase decisions such as poverty, number of people living in the home, and availability of food outlets. All of these factors contribute to the availability of foods in the home, and therefore, food choice.

While this pilot study determined the feasibility of measuring food inventory at multiple times, there were several limitations. This study was tedious in households where the pantry was unorganized. In homes that did not contain a lot of food items, the inventory was completed in under 30 minutes, but in homes that contained a lot of food items, the inventory took up to 1 hour to complete each time. A limitation of the six-item food security module is the lack of identification of food insecurity among children in the household. In addition, most of the measurements of quantity were estimates, because the exact measurements of certain food items could not be obtained. Furthermore, the results may not represent the general population because of the small sample size (n = 9), which was underpowered for a careful examination of factors associated with weekly variation. Finally, future work will need to link household availability, using multiple HFIs, with dietary intake.

Despite the noted limitations to this study, there were a number of strengths. A notable success was the ability to recruit and retain all participants throughout all parts of the project. The results of this study emphasize the importance of multiple home assessments, using direct observation. It is evident that a single point of data collection does not provide an accurate representation of usual foods present in the home. In addition, most homes were not visited on the same day of the week, which provided a better understanding of usual availability. Income cycles were described with the collection of the demographic information. Since 50% of the participants received income every 2 weeks, this variation was captured throughout the 30 days of data collection. The number of home observations that should be conducted over the month has yet to be determined. It is evident a single measurement does not suffice, but more research should be done in order to determine the number of times household food inventory should be conducted, and the frequency.

The availability and accessibility of certain foods within the home has been strongly associated with food choice [10]. This study examined food availability by conducting multiple in-home assessments over the course of one month. Weekly availability of household food items was captured by modifying an existing household food inventory instrument, and recruiting and retaining a sample of nine households. The findings from this study add to the body of research on household food availability by providing detailed information on monthly variability.

## Competing interests

The authors declare that they have no competing interests.

## Authors' contributions

CS and JRS developed the original idea for assessing household food availability. JRS worked with CS on the development of the instrument and the protocol for collection of data. CS wrote the first draft of the paper. CS, JRS, AM, and JA read and approved the final manuscript.
